# A multicentric study on stigma towards people with mental illness in health sciences students

**DOI:** 10.1186/s12909-021-02695-8

**Published:** 2021-06-07

**Authors:** Ana Masedo, Pamela Grandón, Sandra Saldivia, Alexis Vielma-Aguilera, Elvis S. Castro-Alzate, Claudio Bustos, Cristina Romero-López-Alberca, J. Miguel Pena-Andreu, Miguel Xavier, Berta Moreno-Küstner

**Affiliations:** 1 Department of Personality, Evaluation and Psychological Treatment, Universidad de Málaga, Málaga, Spain; 2 MARISTAN Network, Málaga, Spain; 3 Department of Psychology, Universidad de Concepción, Concepción, Chile; 4 Department of Psychiatry and Mental Health, Universidad de Concepción, Concepción, Chile; 5 Doctorate in Mental Health. Faculty of Medicine, Universidad de Concepción, Concepción, Chile; 6 Human Rehabilitation School, Universidad del Valle, Cali, Colombia; 7 Department of Psychology, Universidad de Cádiz, Campus Río San Pedro, 11519 Puerto Real, Cádiz Spain; 8 Centro de Investigación Biomédica en Red de Salud Mental (CIBERSAM), Instituto de Salud Carlos III, Madrid, Spain; 9 Department of Public Health and Psychiatry, Universidad de Málaga, Málaga, Spain; 10 Comprehensive Health Research Centre, NOVA Medical School, Lisboa, Portugal; 11 Biomedical Research Institute of Málaga (IBIMA), Málaga, Spain

**Keywords:** Stigma, Undergraduate education, Health sciences, Attitudes, Mental health

## Abstract

**Background:**

There is evidence of negative attitudes among health professionals towards people with mental illness but there is also a knowledge gap on what training must be given to these health professionals during their education. The purpose of this study is to compare the attitudes of students of health sciences: nursing, medical, occupational therapy, and psychology.

**Methods:**

A comparative and cross-sectional study in which 927 final-year students from health sciences university programmes were evaluated using the Mental Illness: Clinicians’ Attitudes (both MICA-2 and MICA-4) scale. The sample was taken in six universities from Chile and Spain.

**Results:**

We found consistent results indicating that stigma varies across university programmes. Medical and nursing students showed more negative attitudes than psychology and occupational therapy students in several stigma-related themes: recovery, dangerousness, uncomfortability, disclosure, and discriminatory behaviour.

**Conclusions:**

Our study presents a relevant description of the attitudes of each university programme for education against stigma in the formative years. Results show that the biomedical understanding of mental disorders can have negative effects on attitudes, and that education based on the psychosocial model allows a more holistic view of the person over the diagnosis.

## Background

Stigma towards people with Severe Mental Disorders (SMD) is a cross-cutting issue among different groups of healthcare professionals at both primary and secondary levels, specifically nurses [1], doctors [2], occupational therapists, and psychologists [3]. Evidence suggests that stigma must be addressed in the first years of training in the early years of professional studies [4, 5]. It has also been found that, as students advance through their university programme, their attitudes harden and become more resistant to change [6]. We have designed a study for this population – health science students – in this situation, in order to explore negative attitudes related to stigma. Specifically, the main university professions that are present in mental health teams are graduates in psychology, occupational therapy, medicine, and nursing students.

Nursing professionals have often reported their insecurity with regards to treating mental illness. Weare et al. [7] reported that intensive-care nurses felt that they were unprepared to care for patients with mental health disorders; in this sense they reported a need for further training and education. Available evidence demonstrates that medical students typically hold negative attitudes towards psychiatric or mental health disorders [8]. Moreover, a few studies have reported that psychiatrists can tend to have a more negative attitude towards individuals with mental illness than a sample of the general population [9]. Following other empirical studies, an expression of stigma in future psychiatrists consists of negative stereotypes about the personality and capacities of people suffering from mental disorders, the feeling that the recovery expectation of severe mental illness patients is non-existent, as well as a perception that people with schizophrenia are unpredictable and dangerous to the public [10].

On the one hand, research showed that psychology students were significantly less comfortable treating schizophrenia, not to mention being dissatisfied with their current training in SMD [11]. According to previous research, psychology students did not differ from medical students at the baseline of experimental intervention, with regard to their mean stereotypes scores [12]. Equally, the majority of psychology students believed that people with a mental health condition were unpredictable, antisocial, and dangerous [13]. Some authors have noted that psychology students, who favoured biogenetic explanations over psychosocial ones, reported less attribution of responsibility and more pessimistic perceptions of prognosis. Further, it has been argued that biogenetic explanations might adversely affect recovery [12, 14] and lead to further scepticism about the usefulness of psychosocial treatments of schizophrenia [14].

Despite the attitudes of occupational therapy students not having been studied in depth, we present some updates of studies in Latin America [15, 16] that have affirmed that occupational therapists still have a stigmatising attitude towards individuals with a mental health diagnosis.

There are fewer studies on stigma in students of psychology and occupational therapy than in medical and nursing students. In this regard, there is a knowledge gap on what training must be given to health professionals during their education. In addition, despite it being well known that medical and nursing students hold beliefs about the dangerousness of people with mental health difficulties and have pessimistic perceptions of prognosis, little is known about how comfortable they feel in treating or diagnosing mental illnesses, how this overshadows negative attitudes, how they feel about disclosing mental health disorders, or their attitudes towards the mental health field.

To determine priorities in the educational needs of future mental health professionals, comparative studies may throw light on what may be the relevant stigma-related themes and the future lines of intervention against stigma. With a view to bridging this knowledge gap, we designed a study to detect negative attitudes in health students. Therefore, the main aim of this study is to compare the attitudes of final-year students of health sciences: in particular, nursing, medical, occupational therapy, and psychology students.

## Methods

### Design

This study has been designed with a multicentric, and cross-sectional sample.

### Sample

Students attending regular classes in their last years of the university programme (medicine, nursing, psychology, and occupational therapy) made up the sample. We specifically selected students in the year just before they started their shift practices, practicum, and internships in mental health within the framework of the university programme and academic centre and we invited them to participate in the study. Participation was voluntary.

A total of 48 (4.92%) students declined to participate, leaving 927 health students who correctly completed all questionnaires, 457 from Spain (55.1% females) and 470 from Chile (62.7% females) At Universidad de Malaga, 9.09% (*n* = 10) of nursing students declined to participate, while in psychology it was 5.15% (*n* = 5), 12.00% (*n* = 3) in occupational therapy and 0.6% (*n* = 1) for medical students. In Universidad de Cadiz, all those students invited to the study participated, with the exception of those who did not attend the day the information was collected. In Chile, at Concepcion University 7.29% (*n* = 10) of nursing students and 5.55% (*n* = 2) of psychology did not participate. In Universidad Andres Bello and Universidad Las Americas, everyone agreed to participate and in Universidad San Sebastian, only 5.00% (n = 1) did not accept the invitation to participate in the study. Fifteen Chilean medical students (6.12%) decline to participate in the study.

### Setting

Six universities were used as sites, two from Spain and four from Chile, and 927 students were recruited. In Spain, the final sample size by university programme was: psychology (*n* = 109), nursing (*n* = 149), occupational therapy (*n* = 22), and medicine (*n* = 177,); in Chile, sample size by university programme was: psychology (*n* = 34), nursing (*n* = 177), occupational therapy (*n* = 29) and medicine (*n* = 230).

### Questionnaires description

#### Attitudes towards mental illness and psychiatry

The Mental Illness Clinicians’ Attitudes (MICA-2) scale was applied to medical students, and a later version, MICA-4, was used with the other health-related university programmes. The MICA-2 16-item scale was developed with input from mental health service users, carers, medical students, and trainee psychiatrists, and was tested using third year english medical students medical students [17]. MICA-4 is the slightly changed version designed to assess stigma in mental health students (though not psychiatric) involved in the multidisciplinary team which is needed for the mental health field. The scale was tested on nursing students in their foundation year of an English university [18].

Both scales MICA-2 and MICA-4 have 14 items which are equivalent and are comparable. The anchor points are 1–6 from strongly agree to strongly disagree. A higher score indicates a higher stigmatisation or more negative attitudes, and each participant obtains a score within a range of 16–96 points.

The attitudes underlying MICA follow certain themes: how students see psychiatry and mental health; the recovery of people with mental illness; the dangerousness of people with mental illness; how comfortable students feel around people with mental illness; disclosure of their own mental health experiences diagnostic overshadowing, and discriminatory behaviour towards people with mental illness.

Previous psychometric testing of the scale has shown good internal consistency, with Cronbach’s alpha of ɑ = 0.79 [17] and ɑ = 0.72 [18], and test-retest reliability (concordance) was 0.80 (95%CI: 0.68–0.91) [17]. For our total sample, acceptable levels of internal consistency were found, with ɑ = 0.65 for total sample. An analysis by programme found in Chile ɑ = 0.65 for nursing, ɑ = 0.63 for medicine, ɑ = 0.57 for occupational therapy and ɑ = 0.29 for psychology. In Spain, internal consistency was ɑ = 0.67 for nursing, ɑ = 0.56 for medicine, ɑ = 0.67 for psychology and ɑ = 0.62 for occupational therapy. So, besides psychology in Chile, the reliability of the test is adequate for all groups.

#### Socio-demographic questions

Socio-demographic questionnaire included questions and information about previous contact with individuals with a diagnosis of a mental health disorder and the course or semester they were studying at the moment of assessment. “Previous contact” refers to any contact with persons with mental health disorder, for example personal experience (to have family or friends with mental health disorders o also work experience).

### Procedure 

Students were asked to respond to socio-demographicquestions  either MICA-2 (medical students) or MICA-4 (other health students), according to their studies. Data were collected between October 2015 and March 2016.

Approval to conduct this study was granted by the ethics committee of the principal investigator’s university in Spain and Chile.

### Data analysis

Descriptive statistics were used with socio-demographic variables. Multivariate non-parametric analyses based on ranking [19] were carried out to determine if there were differences between the student groups in the following variables: university programme, gender, previous contact, and country. The post hoc Tukey test was applied to study any differences between the university programmes included in the study. Finally, we applied the c^2^ test to explore respondents’ attitudes on the main areas of the questionnaire.

## Results

The sample consisted of 927 health students. Participants were mostly female (59%), with a mean age of 23.16 (SD: 2.86), medical students (43.90%), and who had not had any previous contact with people with mental illness (81.98%). Detailed frequencies of the variables – gender, previous contact, university programme, and country – are displayed in Table 1.

**Table 1:** Descriptive variables of the sample

	Chile	Spain	TOTAL
***Gender***			
Male	218 (46.38%)	162 (35.44%)	380 (41%)
Female	252 (53.62%)	295 (64.55%)	547 (59%)
***Previous contact***			
Yes	94 (20.09%)	69 (15.16%)	163 (17.58%)
No	374 (79.91%)	386 (84.84%)	760 (81.98%)
DK/NA			4 (0.43%)
***University Programme***			
Medicine	230 (48.93%)	177 (38.73%)	407 (43.90%)
Nursing	177 (37.65%)	149 (32.6%)	326 (35.6%)
Occupational Therapy	29 (6.17%)	22 (4.81%)	51 (5.50%)
Psychology	34 (7.23%)	109 (23.85%)	143 (15.42%)
***Age***			
Mean (SD)	23.16 (2.84)		

Table 2 lists the multivariate test results, showing significant *p* values in the total scores of MICA (versions 2 and 4) for the following variables: university programme (*F* = 11.66, *p* < 0.001), country (*F* = 17.57, *p* < 0.001), gender (*F* = 11.66, *p* < 0.001), and previous contact (*F* = 5.07, *p* < 0.001). As can be seen in the table, higher scores indicate that there was more stigmatisation by males (compared to females) and by students who had not been in previous contact with people with a mental disorder.

**Table 2:** Results of multivariate testing on the MICA scale in the complete sample

Variable	MICA score (mean ± SD)	*F* (*p*)
*University programme*		11.1 (< 0.001)
Medicine	40.17 ± 6.84	
Nursing	39.07 ± 7.44	
Occupational therapy	32.98 ± 5.96	
Psychology	34.93 ± 6.82	
*Gender*		11.66 (< 0.001)
Male	40.37 ± 7.06	
Female	37.67 ± 7.33	
*Previous contact*		5.07 (< 0.001)
Yes	36.42 ± 7.91	
No	39.05 ± 7.14	

When analyzing the total MICA score using multiple regression, considering career, country, country program, previous contact, sex and age as predictors, a statistically significant model results, F (10,910) = 14.4, *p* < 0.001, which meets the assumptions of linearity, residual normality, and homoscedasticity. Specifically, as observed in Table 3, the coefficients were significant for the differences between Spain and Chile (b = -1.82, *p* = 0.02), between nursing with psychology (b = -7.1, *p* < 0.001) and occupational therapy (b = -5.42, *p* < 0.001). There were also differences between gender (b = -1.33, *p* = 0.01), and presence of contacts (b = -2.1, *p* <0.001). On the other hand, when analyzing the model by careers, significant differences were found in the Psychology career between Chile and Spain (b = 5.07, *p* <0.001).

**Table 3:** Multiple regression model for prediction of MICA-2 and MICA-4

Coefficients	Estimate	Std. Error	t value	*p*-value
Constant	41.32	0.69	60.03	< 0.01
Country = Spain	− 1.82	0.77	− 2.37	0.02
program = Medicine	0.74	0.74	1.01	0.31
program = Psychology	−7.1	1.3	−5.48	< 0.01
program = Ocupational Therapy	−5.42	1.39	−3.89	< 0.01
Age (years)	0.14	0.08	1.74	0.08
Gender = Female	−1.33	0.53	−2.5	0.01
Previous contact = Yes	−2.1	0.61	−3.47	< 0.01
Country = Spain X program = Medicine	−0.5	1.05	−0.47	0.64
Country = Spain X program = Psychology	5.07	1.56	3.24	< 0.01
Country = Spain X program = Ocupational Therapy	−0.52	2.1	−0.25	0.8

Note: The constant represents the MICA level for the nursing career in Chile of a 23.16-year-old male. With no contact with people with mental health disorders

Multiple regression analysis shows significant differences in programs between countries.

The post hoc Tukey test results comparing university programmes revealed significant differences between psychology and nursing (*F* = 4.142, *p* < 0.001), between psychology and medicine (*F* = 5.239, *p* < 0.001), between occupational therapy and nursing (*F* = 6.092, *p* < 0.001), and between medicine and occupational therapy (*F* = 7.188, *p* < 0.001).

The post hoc Tukey test and total average scores attained in MICA by university programme and country are shown in Table 4. Results suggest that four groups could be distinguished according to the level of stigma:

(A) Group 1, with very high values, comprised of medicine and nursing students in Chilean universities.
(B) Group 2 comprised of nurses in Chilean universities (which is halfway between the mean of groups A and B) and medicine and nursing students in Spanish universities.
(C) Group 3 comprised of nursing and psychology students in Spanish universities.
(D) Group 4, with the lowest average, comprised of occupational therapy and psychology students in Chilean universities.

**Table 4:** Results from post hoc Turkey test comparison between university programme and country, distinguishing four groups according to level of stigma (A = highest stigma, D = lowest stigma)

C = Chile S = Spain	Group	MedC	NurC	MedS	NurS	PsyS	OTC	PsyC	OTS
MedC	A		0.574	0.010	0.000	0.000	0.001	0.000	0.000
NurC	A, B			0.771	0.213	0.027	0.000	0.000	0.013
MedS	B				0.979	0.009	0.006	0.012	0.000
NurS	B, C					0.162	0.045	0.000	0.003
PsyS	C, D						0.846	0.142	0.275
OTC	D							0.988	0.987
PsyC	D								1
OTS	D								

Finally, Table 5 displayed c^2^ tests used to explore differences between specific attitudes relating to themes.

**Table 5:** University programs’ statistical differences based on U Mann Whitney test item by item of MICA-2 vs MICA-4

	University program					
ITEMS	Medical students	Nursing students	Psychology students	Occupational Therapy students	χ^2^	***P*** value
*Original ítem MICA 2 [20] **Differences MICA 2 and MICA-4						
1. I just learn about psychiatry because it is in the exam and would not bother reading additional on it*	2.92 (1.328)	2.773 (1.326)	2.021 (1.265)	2.471 (1.206)	(3) = 67.12	< 0.001
Aprendo sobre salud mental simplemente cuando tengo que hacerlo y ni me molestaría en leer material adicional sobre el tema.						
** MICA-2 uses the word psychiatry and MICA-4 uses the word mental health						
2. People with severe mental illness can never recover enough to have a good quality of life *	2.243 (1.018)	2.193 (1.057)	1.763 (0.826)	1.588 (0.638)	(3) = 41.72	< 0.001
Las personas con un trastorno mental grave nunca podrán recuperarse lo suficiente como para tener una buena calidad de vida.						
**No differences between MICA 2 and MICA-4						
3. Psychiatry is just as scientific as other fields of medicine*	2.206 (1.351)	1.255 (0.826)	1.155 (0.781)	1.216 (0.756)	(3) = 222.28	< 0.001
Trabajar en el campo de la salud mental es tan respetable como trabajar en otros campos de salud y cuidado social.						
** In MICA-4 psychiatry and medicine are substituted by the words: mental health field and health’ field						
4. If I had a mental illness, I would never admit this to my colleagues for fear of being treated differently*	2.722 (1.205)	2.84 (1.233)	2.423 (1.049)	2.255 (1.129)	(3) = 15.04	0.0017
Si tuviera un trastorno mental, no lo admitiría nunca frente a ninguno de mis amigos porque temería ser tratado de manera diferente.						
**MICA-2 and MICA-4 are the same						
5. People with mental illness are dangerous more often than not*	2.845 (1.043)	2.969 (1.131)	2.01 (0.994)	2.388 (0.975)	(3) = 74.13	< 0.001
Las personas con un trastorno mental grave son a menudo peligrosas.						
** MICA-2 and MICA-4 are the same						
6. Psychiatrists know more about the lives of people they treat with mental illness, compared to their careers (family members or friends of people with mental illness)*	3.418 (1.235)	3.445 (1.304)	2.959 (1.33)	2.686 (1.225)	(3) = 24.15	< 0.001
Los profesionales sanitarios y de servicios sociales saben más sobre la vida de las personas tratadas por un trastorno mental que los propios miembros de la familia o amigos.						
**MICA 4 uses Health Professionals instead of psychiatrists						
7. If I had a mental illness, I would never admit this to my colleagues for fear of being treated differently *	3.534 (1.226)	3.393 (1.192)	3.073 (1.242)	2.569 (1.237)	(3) = 32.74	< 0.001
Si tuviera un trastorno mental, no lo admitiría nunca frente a mis compañeros de trabajo por temor a ser tratado de manera diferente.						
**Not differences between MICA-2 and MICA-4						
8. Being a psychiatrist is not like being a real doctor*	1.667 (0.957)	1.864 (1.178)	1.833 (1.167)	1.843 (1.027)	(3) = 4.66	0.198
Ser un profesional sanitario o social en el área de salud mental no es como ser un profesional sanitario o social real.						
**MICA 4 uses “health professional” instead of psychiatrist and doctor						
9. If a consultant psychiatrist instructed me to treat people with mental illness in a derogatory manner, I would not follow the consultant’s instructions*	1.573 (1.12)	1.662 (1.371)	1.423 (1.039)	1.588 (1.344)	(3) = 4.51	0.211
Si un compañero de trabajo con mucha experiencia me dijera que tratase a las personas con un trastorno mental de manera irrespetuosa, no seguiría sus instrucciones.						
**Consultant psychiatrist is substituted by “work colleague with experience” in the version MICA-4						
10. I feel as comfortable talking to a person with mental illness as I do talking to a person with physical illness*	3.28 (1.213)	2.941 (1.193)	2.458 (1.256)	2.333 (1.125)	(3) = 58.73	< 0.001
Me siento tan cómodo hablando con una persona con un trastorno mental como con una persona con enfermedad física.						
**MICA 2 and MICA-4’item 10 is the same						
11. It is important that any doctor supporting a person with mental illness assess the physical health of the person with mental illness*	1.692 (0.931)	1.978 (1.04)	2.062 (1.029)	1.784 (0.610)	(3) = 30.69	< 0.001
Es importante que cualquier profesional que trabaja en salud atendiendo a una persona con un trastorno mental se asegure de que su salud física está siendo evaluada.						
** MICA 2 and MICA-4’item 11 is the same						
12. The public does not need to be protected from people with mental illness*	3.661 (1.255)	3.409 (1.388)	2.722 (1.344)	2.667 (1.143)	(3) = 52.17	< 0.001
La población no necesita ser protegida de personas con un trastorno mental grave.						
**Not differences between MICA-2 and MICA-4						
13. If a person with a mental illness complained of physical symptoms such as chest pain, it is usually part of their mental illness*	2.223 (1.016)	2.209 (0.901)	2.361 (1.072)	2.078 (1.017)	(3) = 2.12	0.548
Si una persona con enfermedad mental refiere síntomas físicos (como dolor de pecho), lo primero que pensaría es que están relacionados con su trastorno mental.						
**Not differences between MICA-2 and MICA-4						
14. General practitioners should not be expected to complete a thorough assessment for people with psychiatric symptoms because they can be referred to a psychiatrist*	2.305 (1.092)	2.592 (1.275)	2.865 (1.448)	2.571 (1.458)	(3) = 18.56	< 0.001
No debe esperarse que los médicos generales realicen una evaluación exhaustiva de las personas con síntomas psiquiátricos porque éstas pueden ser derivadas a un psiquiatra.						
**Not differences						
15. I would use the term “crazy”, “nutter”, “mad”, etc., to describe people with mental illness that I have seen on the ward, to colleagues*	1.98 (1.125)	1.752 (1.018)	1.546 (0.750)	1.51 (0.880)	(3) = 23.57	< 0.001
Si tuviera que describir a mis compañeros, a las personas con un trastorno mental que he atendido en mi trabajo, usaría los términos: “loco”, “chiflado”, “chalado”, etc.						
**Not differences						
16. If my colleague told me they had a mental illness, I would still want to work with them*	1.865	1.804 (0.865)	1.753 (0.93)	1.451 (0.642)	(3) = 17.89	< 0.001
Si un compañero/a de trabajo me dijera que tiene un trastorno mental, seguiría queriendo trabajar con él/ella.						
**Not differences						

Regarding attitudes and interest in psychiatry or mental health themes, medical students disagree more with the sentence ‘Working in the field of psychiatry is as respectable as working in other fields of health’ than the rest of health students, and agree more with the sentence ‘I learn about mental health when I have to (an exam), and I would not even bother reading additional material on the subject’, followed by nursing, occupational therapy, and psychology students, who showed the lowest score. Differences were not significant for Item 8.

In relation to the theme of recovery of people with mental illness, differences by university programme were significant for Item 2. Medical and nursing students showed significantly more agreement with the sentence ‘People with severe mental disorders will never be able to recover sufficiently’ than psychology and occupational therapy students.

The theme of MICA Dangerousness of people with mental illness was also analysed. Significant differences were found for Items 5 and 12. Medical and nursing students showed more agreement with the sentence ‘People with a serious mental disorder are often dangerous’, followed by occupational therapy students, with psychology students yielding the lowest scores. Medical and nursing students also showed greater disagreement with the sentence ‘The population does not need to be protected from people with severe mental disorders’ than occupational therapy students, and psychology students had the lowest scores.

Regarding the theme of how comfortable students feel being around people with mental illness, differences were significant for Item 10. Medical students disagreed more with the sentence ‘I feel as comfortable talking to a person with mental illness as I do to a person with physical illness’, followed by nursing, occupational therapy, and psychology students.

In relation to the MICA theme regarding disclosure of their mental health experiences*,* differences were significant for Items 4 and 7. Nursing students showed more agreement with the sentence ‘If I had a mental illness, I would never admit this to my friends because I would fear being treated differently’, followed by medical, psychology and occupational therapy students. Medical students, however, showed the highest score for Item 7 (‘If I had a mental illness, I would never admit this to my work colleagues for fear of being treated differently’), followed by nursing, psychology, and occupational therapy students.

Analysis of the theme of diagnostic overshadowing threw up significant differences in Items 11 and 13. Psychology students disagreed more with the sentence ‘It is important that any health professional caring for a person with mental illness should ensure that their physical health is assessed’ than nursing, occupational therapy, and medical students. Psychology students also showed more agreement with the sentence ‘General practitioners should not be expected to conduct a comprehensive assessment of people with psychiatric symptoms because they should be referred to a psychiatrist’ than the rest of the students. In relation to Item 13 (‘If a person with mental illness complained of symptoms [such as chest pain], the first thing I would think is that it is related to their mental health’), there were no significant differences between the students.

Finally, in relation to the theme of discriminatory behaviour towards people with mental illness, we found significant differences in Item 15 but not in Items 9 and 16. Medical students showed the highest score in Item 15 (‘I would use the terms “crazy”, “nutter”, “mad”, etc. to describe people with mental illness that I have seen in the ward to colleagues’), followed by nursing, psychology, and occupational therapy students. Medical and nursing students also showed more disagreement with the sentence ‘If my colleague told me they had a mental illness I would still want to work with them’ than psychology and occupational therapy students.

## Discussion

This study has compared the stigmas of students in their final year of four different health science university programmes in two different countries.

Taking the results as a whole, medical students have reported more negative attitudes than nursing, psychology, and occupational therapy students towards the mental health profession and the direct treatment of patients with psychiatric problems. This is in line with previous studies reporting that medical students regarded clinical work with psychiatric patients as unappealing and stressful [8, 21].

We also analysed stigmatised misconceptions about mental illness, such as the assumption of dangerousness and pessimistic prognoses. Medical and nursing students showed higher stigma in this area than psychology and occupational therapy students [22, 23]. Regarding students’ disclosure of their own mental health problems, we have found that medical and nursing students are more hesitant to talk about their problems. This is consistent with a recent qualitative and narrative study in which the authors strongly affirmed that medical students and doctors often resist seeking assistance for their own psychiatric problems, perhaps due to fear of exposure to stigmatisation [24]. One resource linked to a campaign against stigma is *Coming Out Proud* (COP), a three-week group intervention that offers support to reduce the negative impact of stigma. The COP programme encompasses weighing the costs and benefits of disclosure in deciding whether to come out, considering different strategies for coming out, and obtaining peer support through the disclosure process [25]. Also, it has been probed recently the benefits on mental health (ej depressive symptoms) between those that disclose than those that not disclosure with lower costs program and hence more scaleable [26].

We can affirm that nursing students represent the second group with more negative attitudes. However, the stigma by nursing students from Spanish universities is slightly more favourable, which could be explained by the development of the socio-community model *Opening Doors* and also by the weightier biomedical model in the nursing programme from Chile, in line with international studies [5, 27, 28]. Our results highlight that future efforts in the reduction of stigmatising attitudes in mental health nurses should focus on increasing recovery expectations. This should reduce the misconceptions about violence related to mental health problems, and reducing the stigma associated with disclosure about their own mental health problems to others and encouraging positive attitudes towards people with mental health difficulties.

An unexpected result was that psychology students reported a higher stigmatised perception of the medical needs of people with mental health difficulties, which are sometimes not taken seriously in medical settings [20]. These prejudices have been evidenced in doctors [29] but we found that psychology students showed more disagreement than the other groups in those items related to the need for assessing the physical health of individuals diagnosed with a mental health disorder. Psychologists may minimise physical or medical problems in the same way that psychology programmes tend not to include medical aspects.

Another relevant finding is that psychology students in Spain had worse attitudes than psychology students in Chile than occupational therapy students in both countries. Future studies would need to consider the possible influence of theoretical backgrounds or differential aspects of curricula inherent to each university programme with regard to stigma. Previous studies have affirmed that nursing and medical programmes are mainly based on the biogenetic model and are associated with a more pessimistic view about the recovery of the mentally ill [14, 23]. On the other hand, psychology programmes generally combine a diathesis-stress model with a biogenetic model as a background to psychopathology, and psychology students are educated with the conception of psychological trauma being among the main causes of mental illness. Our results could be interpreted as in line with the findings of a body of research documenting that the biomedical understanding of mental disorders could have negative effects on attitudes related in particular to recovery [30]. Serafini et al. [23] researched whether regarding mental disorders could have negative effects on attitudes towards schizophrenia as a genetic or environmental disorder might influence the perceived beliefs of nurses, doctors, and medical students towards people with schizophrenia. Genetic attributions were associated with higher stigmatisation by the view, perhaps, that the condition was chronic, with no possibility of recovery [23]. Our data seem to support that there are fewer stigmas in psychosocial university programmes (i.e. psychology and occupational therapy), and we conclude that our results show that education based on the psychosocial model allows a more holistic view of the person over the diagnosis—although other alternatives explanations needs to be considered. Recently, it has been evidenced that expectation about of working conditions in psychiatry (what varies from lower to lower-middle income countries) also has a weight on stigma and this also could explain the shortage and poor recruitment of medical students [31].

The strength of this study lies in the inclusion of students from different universities in cities of two Spanish-speaking countries with different cultures. We have not found huge differences between attitudes of students in the same university programmes in either country. Future reports should test the impact of the rotations and practicum on these students’ attitudes. It is also important to consider that the students participating in this study had not yet undergone clinical time in psychiatry at the moment of assessment (e.g. at the beginning of the final year of the university programme). According to other authors, it is possible that having experience with mental illnesses would help students to acquire skills and knowledge of what to expect when working with persons diagnosed with mental health problems and to develop confidence in communicating with them [32]. In this sense, several studies found that contact with people with mental illness can reduce students’ fear, prejudices, and apprehension [33, 34].

### Implications for practice and education

Before direct contact with mental health patients, educational needs should be addressed on the issue of stigma. Firstly, university programmes in medicine and nursing should be addressed as a priority what is coherent with previous research [7–9]. Psychologists represent a group with more moderate stigma but which also needs anti-stigma educational reinforcement. Finally, the group with the best outcomes, comparatively, is occupational therapy.

We would recommend that universities should make changes in their health sciences programmes and provide earlier contact with mental health rotations, not just reserving them for the last year of the health programme. There are implications for teaching in the academic field which could benefit from several recommendations to reduce these negative attitudes. It is crucial to expose health students with negative attitudes to scientific data showing that more than 50% of persons with schizophrenia recover from this disorder [10]. They need to hear real testimonies of individuals who have recovered and, even more so, these testimonies should come from doctors or other health professionals. Secondly, we agree with Magliano et al. [35] about the need for revision of the psychiatric curricula for medical and nursing students and the need to pay greater attention to the integration of psychological and medical aspects in clinical management, education on clinical efficacy of psychosocial interventions, and the sensitisation of students regarding the stigma of mental illness as a public health issue. With this topic, we underline the recommendations of Dixon et al. [36] who said that the presentation of autobiographical cases should allow mental illnesses to be viewed within the context of the individual’s entire life. The possibility of including an ‘holistic person-based speciality’ is also worthy of mention [37].

Definitively, the fight against stigma needs really efficient tools and we also encourage a rethink on the key ingredients of anti-stigma programmes in healthcare [38]. To maximise the effectiveness of anti-stigma intervention, programmes need to emphasise recovery and, more importantly, include multiple types of social contact with recovered patients, favouring contact under equal conditions. The most innovative and challenging initiative may be to use recovered patients with high communication skills to train future mental health professionals [39].

### Limitations

Several limitations of this study should also be noted.

1. The sample was drawn from some comprehensive undergraduate courses and the findings cannot be generalised to every undergraduate course in Europe and Latin America. Having data from two different countries for each university programme is an advantage for this study.
2. Mental disorders or illnesses and severe mental disorders are terms included in the MICA scale and we cannot extrapolate these results to schizophrenia or bipolar disorder in particular. According to Fernando, Deane, & McLeod [22] and Dixon et al. [36], disorders with the highest negative attitude are depression, schizophrenia, drug addiction, and alcohol addiction.
3. We have not selected other programs that also students could work with persons with mental health disorders, for example social work.
4. The scales used have not been previously validated into Spanish.
5. We are aware of a small proportion of the sample that refused participation and we do not know if there are any differences between both samples.

Finally, we want to add two more limitations in relation to the present study that should be considered: (1) the scales used have not been previously validated into Spanish and (2) we are aware of a small proportion of the sample that refused participation and we do not know if there are any differences between both samples.

## Conclusions

Our study presents a relevant description of the attitudes of each university programme for education against stigma in the formative years in two different countries. More specifically, we have found that medical and nursing students generally attained higher scores in MICA than psychology and occupational therapy students. An interesting interaction has also been found between university programme and country. Post hoc analyses shed light on the fact that we can identify four groups that demonstrate differences in stigma towards persons with mental disorders. Medical and nursing students in Chile have the most stigmatising attitudes, followed by their counterparts in Spain. The third group includes nursing and psychology students in Spain, with no differences in stigma between the two. Finally, psychology and occupational therapy students have the lowest stigma scores. In conclusion based on our results, we could affirm that there is a priority for education against stigma in the population of students of nursing and medicine programmes.

## Data Availability

The datasets used and/or analysed during the current study are available from the corresponding author on reasonable request.

## Abbreviations

SMD: Severe Mental Disorders
MICA: Mental Illness Clinicians’ Attitudes
